# An Easy Phylogenetically Informative Method to Trace the Globally Invasive *Potamopyrgus* Mud Snail from River’s eDNA

**DOI:** 10.1371/journal.pone.0162899

**Published:** 2016-10-05

**Authors:** Laura Clusa, Alba Ardura, Fiona Gower, Laura Miralles, Valentina Tsartsianidou, Anastasija Zaiko, Eva Garcia-Vazquez

**Affiliations:** 1 Department of Functional Biology, University of Oviedo, C/ Julian Claveria s/n 33006, Oviedo, Spain; 2 USR3278-CRIOBE-CNRS-EPHE-UPVD, Laboratoire d’Excellence “CORAIL”, Université de Perpignan CBETM, 58 rue Paul Alduy, 66860, Perpignan Cedex, France; 3 Coastal and Freshwater Group, Cawthron Institute, 98 Halifax Street East, 7010, Nelson, New Zealand; 4 Marine Science and Technology Centre, Klaipeda University, H. Manto 84, LT-92294, Klaipeda, Lithuania; University of Houston, UNITED STATES

## Abstract

*Potamopyrgus antipodarum* (New Zealand mud snail) is a prosobranch mollusk native to New Zealand with a wide invasive distribution range. Its non-indigenous populations are reported from Australia, Asia, Europe and North America. Being an extremely tolerant species, *Potamopyrgus* is capable to survive in a great range of salinity and temperature conditions, which explains its high invasiveness and successful spread outside the native range. Here we report the first finding of *Potamopyrgus antipodarum* in a basin of the Cantabrian corridor in North Iberia (Bay of Biscay, Spain). Two haplotypes already described in Europe were found in different sectors of River Nora (Nalon basin), suggesting the secondary introductions from earlier established invasive populations. To enhance the surveillance of the species and tracking its further spread in the region, we developed a specific set of primers for the genus *Potamopyrgus* that amplify a fragment of 16S rDNA. The sequences obtained from PCR on DNA extracted from tissue and water samples (environmental DNA, eDNA) were identical in each location, suggesting clonal reproduction of the introduced individuals. Multiple introduction events from different source populations were inferred from our sequence data. The eDNA tool developed here can serve for tracing New Zealand mud snail populations outside its native range, and for inventorying mud snail population assemblages in the native settings if high throughput sequencing methodologies are employed.

## Introduction

Human-mediated translocations of marine organisms have become a widely acknowledged global environmental issue nowadays [1, 2]. Maritime activities like merchant shipping or yachting aid the spread of many species out of their native distribution range, and global change may facilitate the success of exotic species in recipient ecosystems until they become invasive with adverse effects on environment and economies [3, 4]. A successful invader must exhibit a set of differential features [5] allowing passing the different steps of the invasion process and involved barriers: transportation, establishment and spread [3, 6]. Such species usually become of a particular concern for environmental managers and interest for researchers studying patterns in biological invasions.

*Potamopyrgus antipodarum* (New Zealand mud snail) is one of the extremely successful invaders in aquatic ecosystems worldwide. This ovoviviparous prosobranch is currently found in Australia [7], Asia [8–10], Europe [11, 12] and North America [13–15].

Being extremely tolerant, *P*. *antipodarum* is a good candidate to survive the transportation to a new region. The presumed vector of its initial transoceanic introduction to Europe and USA is ballast water [13]. Its further spread within the region could be aided by aquaculture (e.g. translocation of stock or equipment), fisheries (e.g. with boats or gear), recreational activities (e.g. with angling gear or pets) [16] or by natural vectors such as birds or fish [14, 17, 18].

Once it reaches the new region, it can colonize and adapt to a wide range of habitats: estuaries [14, 15], lakes [19], rivers [20], saltwater [21] and even open seas [22]. This mud snail competes with native invertebrates for resources in invaded habitats dominating the invertebrate communities [16, 23]. For example, it has caused the decrease of *Pyrgulopsis robusta* population in USA [24] and the decline of native benthos density and diversity in Poland [25]. They consume up to 75% of primary production, leading to altered nitrogen and carbon cycles in invaded ecosystems [26, 27]. It has been found to resists the impact of parasites [11], and also that of potential predators because it is a poor and often indigestible food for salmon and other fish species [17]. Moreover, Sanderson *et al*. [28] suggested that non-indigenous species like *P*. *antipodarum* are threatening the conservation of endangered salmon due to the alterations they cause in the trophic chain. Due to extremely fast population growth rate it can reach high densities in a short time after incursion, reducing the opportunities for control and mitigation measures. Therefore, early detection is in this case crucial for the efficient rapid response and prevention of the further invasion.

In the last few years, the use of environmental DNA has become a promising tool to detect and survey invasive species in aquatic ecosystems. This method seems to be more sensitive and efficient than traditional surveillance approaches, like visual detection, and does not disturb the aquatic fauna [29–31]. The use of specific primers on eDNA has been successfully demonstrated for a number of species. Examples are fish *Petromyzon marinus* and *Salmo trutta* [32], molluscs such as *Rangia cuneata* in the Baltic Sea [33] and *Xenostrobus securis* in North Spain [34], and others. *Potamopyrgus antipodarum* has also been detected previously directly from water samples [35], as presence-absence based on positive or negative PCR amplification of a fragment of the cytochrome b gene.

Städler *et al*. [36] suggested that the origin of European *Potamopyrgus antipodarum* is located in New Zealand. They found only two haplotypes of 16S rDNA across all Europe shared with snails from the North Island of New Zealand. The marked divergence among the two European haplotypes implies successful colonization by two distinct mitochondrial lineages.

The aim of this study was to demonstrate a cost-effective surveillance strategy for the species and to explore its invasion history in the North Iberian region. We developed specific primers for *Potamopyrgus* based on 16S rDNA sequences, for detecting this mud snail and inferring its lineage directly from water samples.

## Materials and Methods

### The species studied

*Potamopyrgus antipodarum* is small in invaded regions (6–7 mm size in average), but can grow up to 12 mm in its native range (New Zealand). It has a solid operculum and an elongated shell [37]. It is capable to survive in a great range of environmental conditions: salinities 0–38 PSU [38–40], water temperatures 0–28°C [41], and can even resists short times of desiccation [3, 42]. Non-native populations are generally parthenogenetic, consisting almost exclusively of females [3]. One adult in a new habitat can produce an average of 230 juveniles per year [13]. This high reproductive capacity helps *Potamopyrgus* to establish and disperse quickly in a new area. Indeed this capacity is the main reason for the large ecological impact of *P*. *antipodarum*. Even a single individual can result in a massive invasion just in a few months.

### Field sampling

River Nora (Asturias, north of Spain) is a tributary of the River Nalon basin, in the central Bay of Biscay region, of 67 km long and with an average discharge of 20.98 m^3^/s. It is completely isolated from downstream by an impassable barrier and a reservoir for hydroelectric power supply (Priañes dam, 43°23′02″N 5°58′26″W) built in 1953. In February-March 2015, mud snails were sampled from three sites within the River Nora, separated from each other by three kilometers. From upstream to downstream, the sites were: Colloto (coordinates 43.379283, -5.788667); Lugones (coordinates 43.401321, -5.822816); and San Claudio (coordinates 43.382938, -5.931142). Ecological conditions were very similar in all sampling sites, with a bottom of stones and gravel, shallow depth, and resembling water flow.

The sampling protocol was the following: a 1m^2^ quadrat was randomly selected, and all present *Potamopyrgus* individuals were manually collected from the stones (including the underneath sides). This was done simultaneously by three researchers from each site, thus three replicates of 1m^2^ (approx.) were obtained per site. The average number of individuals per replica is a rough but comparable proxy of the density of the *Potamopyrgus* population present in each site. Additionally three liters of water were collected with sterile bottles from the same sampling locations before the search of *Potamopyrgus* individuals.

As negative field controls one liter of water was taken from Llanes beach (seawater), coordinates 43.420461, -4.752003 and mainstream River Nalon (freshwater), coordinates 43.180926, -5.341015. No *Potamopyrgus* individuals were found in these sites despite intensive exploration. No specific permissions were required for sampling in these locations. The River Nora is not within a national park or other protected area. It is of public access. The species *Potamopyrgus antipodarum* is not native from Spain. Moreover it is listed in the register of invasive species (Spanish Directive of 4 August 2013).

### DNA extraction

From tissue samples DNA was extracted with mollusc DNA Kit (Omega Bio-Tek, USA) following the instructions provided by the manufacturer.

1 L of the water samples was filtered using the Supor^®^-200 Membrane Filter (Pall Corporation) with 0.2 μm pore size. The filtration apparatus was cleaned with 10% bleach, rinsed with distilled water and autoclaved between each sampling site. DNA was extracted with the PowerWater^®^ DNA Isolation Kit (Mobio laboratories). The filtration process and eDNA extractions were done under sterile conditions, in a laboratory unit where there was no other tissue samples, to avoid any contamination of the environmental DNA. eDNA extractions also were done inside a PCR laminar flow cabinet prior to extractions treated with ultraviolet light. Blanks containing only water were used as controls in DNA extraction, to confirm that contamination did not occur in the process.

### Design of specific primers

The 16S rRNA gene was chosen for the design of the primer, based on reference nucleotide sequences of 16S rDNA from GenBank plus the sequences obtained in the laboratory from *Potamopyrgus* samples of different origins. Sequences of this gene (either individual 16S DNA sequences or complete mitochondrial genomes), available for *Potamopyrgus* and other mollusk species were downloaded and aligned with the ClustalW application included in BioEdit [43]. Polymorphisms were analyzed with the DNASP software [44]. The different haplotypes were visualized employing the BioEdit Sequence Alignment Editor software [45]. The universal primers designed by Palumbi *et al*. [46] amplifying a 16S rDNA region of approximately 600 nucleotides were used for species barcoding. A region within these amplicons conserved in the genus *Potamopyrgus* but different in the rest of mollusk species was searched. This region was used to design a *Potamopyrgus* genus-specific reverse primer. As forward primer we used the universal 16SAr from Palumbi *et al*. [46].

### Markers employed and PCR conditions

PCR amplification of 16S rDNA using the universal primers described by Palumbi [46] was done with the following protocol. The amplification reaction was performed in a total volume of 40 μl, including Green GoTaq^®^Buffer 1X, 2.5 mM MgCl_2_, 0.25 mM dNTPs, 1μM of each primer, 0.65 U of DNA Taq polymerase (Promega) and 4 μl of template DNA. PCR conditions were the following: an initial denaturation at 95°C for 5 min followed by 35 cycles of denaturation at 94°C for 1 min, annealing at 55°C for 1 min, extension at 72°C for 2 min and a final extension step at 72° for 7 min. The PCR products were sequenced in the DNA sequencing service Macrogen Europe, and the species identifications were confirmed using the BLAST tool from the NCBI.

PCR amplification of partial 16S rDNA from tissue DNA using the new primers set (the newly designed reverse primer and the universal Palumbi’s forward primer) was performed in a total volume of 20μl with the same conditions above, except for the annealing temperature. We assayed six different annealing temperatures: from 55°C to 60°C for selecting the best one (that provides clean and clear amplification products of the expected size with no extra bands). The assays of annealing temperatures showed that the best results were obtained at 60°C. All the PCR products were visualized in 2% agarose gels with 2.5 μl of SimplySafe^™^.

PCR amplification of a fragment of 16S rDNA from the bulk DNA extracted from water samples (eDNA) with the specific primer was performed in a total volume of 20 μl, including Green GoTaq^®^Buffer 1X, 2.5mM MgCl_2_, 0.25mM dNTPS, 1μM of each primer, 6 μl of template DNA, 200ng/μl of BSA (bovine serum albumin) and 0.65 U of DNA Taq polymerase (Promega). The PCR conditions were the same as described above, at the best annealing temperature, but with 45 cycles instead of 35. Amplification products from water samples were purified with the Agarose-Out DNA purification kit (EUR^®^X) and sequenced by Macrogen service.

The cytochrome oxidase I (COI) gene was amplified from DNA extracted from tissue and water samples using the universal primers for invertebrates designed by Geller *et al*. [47] and following the protocol described therein. The difference between the protocols used for tissue and water DNA was the number of cycles in the PCR– 35 and 45 respectively. Negative controls containing only PCR reagents and distilled water were added in every PCR.

### Marker validation

The new primer was first tested *in silico* by an alignment with the BLAST tool in the NCBI database [48].

Adult individuals of brackish and freshwater mollusks (five per species) were collected for testing *in vitro* possible cross-species amplification of the new primer (Table 1). PCR amplification with the universal primers of Palumbi *et al*. [46] was done.

**Table 1:** Adult mollusks sequenced in this study for 16S rRNA and cytochrome oxidase I genes.

Species	Habitat	Origin	Common name	Collection site
*Potamopyrgus antipodarum*	freshwater, brackish	non- native	New Zealand mudsnail	Nora River
*Mytilus galloprovincialis*	marine	Spanish native	Mediterranean mussel	Aviles estuary
*Mytilus trossulus*	marine	non-native	Foolish mussel	Baltic Sea
*Ruditapes philippinarum*	marine	non-native	Japanese carpet Shell	Aviles estuary
*Xenostrobus securis*	brackish	non-native	Axe-head mussel	Aviles estuary
*Mya arenaria*	marine	non-native	Soft-shell clam	Baltic Sea
*Crassostrea gigas*	marine	non-native	Giant oyster	Aviles estuary
*Tylomelania kuli*	freshwater	non-native	Sulawesi snail	pet shop
*Tylomelania toradjarum*	freshwater	non-native	Sulawesi snail	pet shop
*Neritina canalis*	brackish	non-native	Nerite	pet shop
*Neritina punctulata*	freshwater	non-native	Nerite	pet shop

Bivalves and gastropods (five individuals per species) employed for the evaluation of cross-amplification of the specific primers. The origin (native or non-native) is given in relation with Spanish waters.

The new set of specific primers was assayed on DNA extracted from eleven mollusk species described in Table 1.

The sensitivity of the specific primers was determined *in vitro* with serial dilutions of *Potamopyrgus antipodarum* DNA from a known concentration (43μg/ml). PCR amplification and visualization of the PCR product in a 2% agarose gel were performed for each concentration. DNA concentration was measured with a spectrophotometer (SimpliNano^™^ GEHealthcare).

From water eDNA, a fragment of the 16S rDNA was PCR-amplified with the new specific primers set using the protocol described in 2.4. As a positive control, the COI gene was amplified from each eDNA sample as described in 2.4, to test for the quality of the DNA and discard false negatives due to excessive DNA degradation, inhibitors or other reasons.

### Validation of negative results

To confirm that the negative results of PCR with the specific primers performed on eDNA samples were true and not produced by any interference or inhibitor present in the template, the subsamples of the Llanes beach eDNA (6μl) were spiked with *Potamopyrgus antipodarum* DNA of two concentrations: 2μl of *P*. *antipodarum* stock DNA (43 μg/ml), and 2μl of the 1:50 000 dilution from the same stock. PCR amplifications were performed in the same conditions as explained before.

### Phylogenetic analysis

*Potamopyrgus* individuals from River Nora and from different locations in New Zealand (as representatives of native populations), were collected and taxonomically classified *de visu* (Table 2). Three different sequences were obtained from these samples: COI gene [47], 16S rRNA gene [46] and partial 16S rDNA amplified with the specific primers set. Additional sequences assigned to *Potamopyrgus* species were downloaded from GenBank. For each gene, the sequences were aligned with the ClustalW application included in BioEdit [43]. The alignment was converted to MEGA file and a phylogenetic neighbor-joining tree was built using MEGA 4.0 [49], with 10000 bootstrapping and the evolutionary distances were computed using the Tamura-Nei method [50].

**Table 2:** *Potamopyrgus* samples, collected from Asturias and New Zealand, sequenced in this study for 16S rRNA and cytochrome oxidase I genes.

Sample	Place	Country	Species
Pa Ast1 01	Colloto- River Nora	Spain	*P*. *antipodarum*
Pa Ast2 01	Lugones- River Nora	Spain	*P*. *antipodarum*
Pa Ast2 02	Lugones- River Nora	Spain	*P*. *antipodarum*
Pa Ast2 02	Lugones- River Nora	Spain	*P*. *antipodarum*
Pa Ast3 01	San Claudio- River Nora	Spain	*P*. *antipodarum*
Pa NZ1 01	Collins River	New Zealand	*P*. *antipodarum*
Pa NZ2 01	Onomalutu River	New Zealand	*P*. *antipodarum*
Pe NZ3 01	Maitai River Site 1	New Zealand	*P*. *estuarinus*
Pe NZ3 02	Maitai River Site 1	New Zealand	*P*. *estuarinus*
Pe NZ3 03	Maitai River Site 1	New Zealand	*P*. *estuarinus*
Pe NZ4 01	Maitai River Site 2	New Zealand	*P*. *estuarinus*
Pe NZ4 02	Maitai River Site 2	New Zealand	*P*. *estuarinus*
Pe NZ4 03	Maitai River Site 2	New Zealand	*P*. *estuarinus*
Pe NZ5 01	Mangroves Matua Rangarawa	New Zealand	*P*. *estuarinus*
Pe NZ5 02	Mangroves Matua Rangarawa	New Zealand	*P*. *estuarinus*
Pe NZ5 03	Mangroves Matua Rangarawa	New Zealand	*P*. *estuarinus*

## Results

### Specific primers

The new specific primer designed *in silico* within the 16S rDNA sequence was:

Reverse primer: 16SPA-R (5’-TCAAAGATTTTGGATCATAGCT-3’).

Using the 16SAr described by Palumbi *et al*. [46]: 16SAr (5’-CGCCTGTTTATCAAAAACAT-3’) as a forward primer and the new 16SPA-R as a reverse primer, a region of 380 nucleotides within the 16S rRNA gene was amplified. The region is located between sites 5350 and 5730 of the *Potamopyrgus antipodarum* mitochondrion complete genome with GenBank accession number GQ996421.1.

### Marker validation

From BLAST assays *in silico*, the new primer retrieved significant alignments, with 100% identity, 100% coverage, 0.018 E-value and score of 44.1, with *Potamopyrgus antipodarum*, *P*. *estuarinus*, *P*. *doci*, *P*. *opidanus*, *P*. *troglodytes* sequences of 16S rRNA gene. The same values were also obtained with 16S rDNA sequences of *Caldicochlea globosa*, an Australian endemic aquatic snail, and several species of the genus *Sororipyrgus* that are Hydrobiidae snails endemic in New Zealand. All these species except for *Potamopyrgus antipodarum* are currently absent from European aquatic ecosystems.

PCR reactions for assessing primers’ specificity discarded cross-amplification with other mollusks assayed in this study (Table 1A). Consistently with *in silico* results, *in vitro* tests showed that the specific primers give positive PCR amplification (amplicons visible in agarose gels; data not shown) on the species listed in Table 1A only from DNA samples of *Potamopyrgus*.

**Fig 1:**
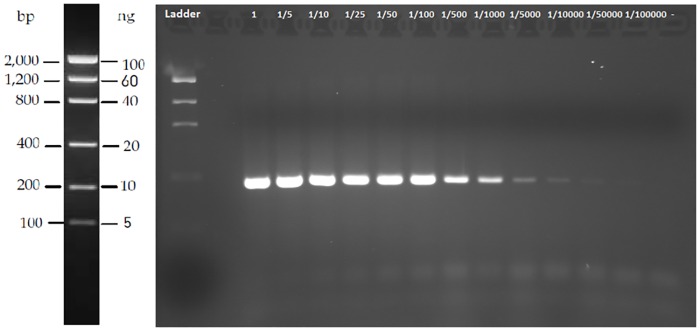
Agarose gel showing PCR amplification products obtained with the species primers’ set for 16S rRNA gene from serial dilutions of *Potamopyrgus antipodarum* DNA (43μg/ml): 1 (no dilution), 1:5, 1:10, 1:25, 1:50, 1:100, 1:500, 1:1000, 1:5000, 1:10000, 1:50000, 1:100000 and a negative control.

The threshold of detection for PCR-visualization in agarose gels was 0.86μg/l, because we obtained a weak but visible band of the amplicon size in the dilution 1 to 1:50 000 from a sample with a concentration of 43μg/ml (Fig 1).

### *Potamopyrgus antipodarum* population in Asturias

In River Nora the *Potamopyrgus antipodarum* population was not identical in the three sampling sites. From the sampling results, the estimated population density was higher in the midstream location of Lugones (Table 3), with 63 individuals/m^2^; meanwhile downstream San Claudio has quite low density of 6 individuals/m^2^. None of the individuals was >7mm. In the downstream site the relative abundance of juveniles (<3mm) was clearly lower than in upstream areas (Table 3).

**Table 3:** *Potamopyrgus antipodarum* specimens collected from different sites within Nora River, classed by size, and total density. The same sampling protocol from three replicates of 1m^2^ was employed in all sites.

Site	Density (individuals/m^2^)	Individuals ≥ 3mm	Individuals < 3 mm
Colloto (upstream)	18	33.3%	66.7%
Lugones (midstream)	63	39.7%	60.3%
San Claudio (downstream)	6	83.3%	16.7%

**Fig 2:**
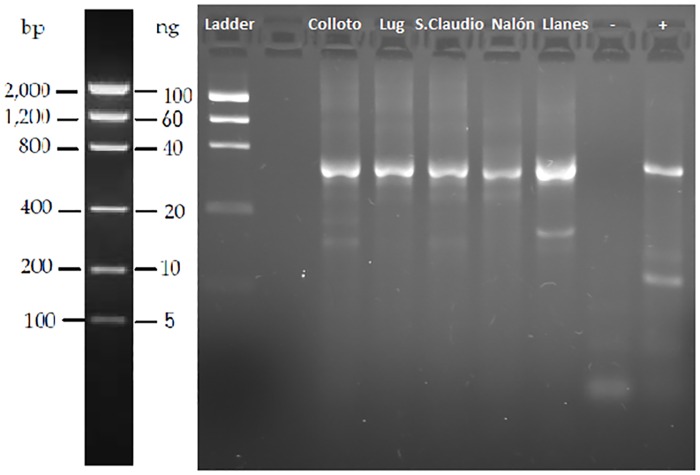
Amplification products of the cytochrome oxidase I gene obtained from PCR with universal primers on water samples. Sampling sites: River Nora: Colloto, Lug (Lugones) and San Claudio, River Nalón and Llanes beach;–and + are negative and positive controls respectively.

**Fig 3:**
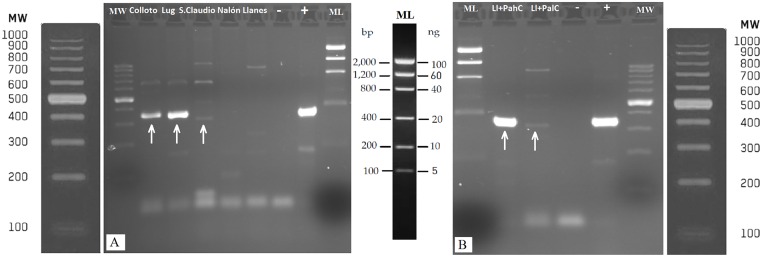
A) PCR products of the partial 16S rRNA gene obtained with the taxon-specific primers, on DNA extracted from water samples of River Nora (Colloto, Lugones and San Claudio sites), River Nalón and Llanes beach. Positive amplifications are marked with an arrow. B) Validation of negative results: amplification products of the same gene obtained from Llanes beach water DNA spiked with *Potamopyrgus antipodarum* DNA. Ll+PahC and Ll+PalC are high and low concentration of *Potamopyrgus antipodarum* respectively (43μg/ml and dilution 1:50000 respectively).–and +, negative and positive controls respectively.

In all eDNA samples obtained from water, PCR with the universal COI primers [47] yielded amplification products of the expected size around 650 nucleotides (Fig 2). The water samples from River Nora provided positive PCR amplification with the taxon-specific primers designed herein (Fig 3A). In the other two control sites, Llanes beach and River Nalón; no amplification was obtained with these primers as expected since *Potamopyrgus* mollusks are not present there. The positive bands observed in agarose gel for River Nora water samples were purified, sequenced and the sequences unequivocally identified as *Potamopyrgus antipodarum*, GenBank accession numbers KU933000-KU933002. The PCR products gave clear chromatograms directly readable, without any trace of nucleotide mixture in any site.

In the agarose gel it can be seen that the band for Lugones is bigger and brighter than for San Claudio location (Fig 3A), concordantly with different population densities. The method can be considered quite sensitive because PCR product was detectable in agarose gel even for San Claudio sample where the observed density was only 6 individuals/m^2^ (Fig 3A).

On the other hand, the negative results obtained from field water samples were confirmed by the additional validation test. Positive PCR amplification from Llanes beach water sample was obtained when *Potamopyrgus* DNA was added (Fig 3B). A clear band was seen in the two mixtures, one of high concentration with an amount of approximately 86 ng of *P*. *antipodarum* DNA and the other of low concentration with approximately 1.72pg of DNA. The positive results obtained in this last PCR indicate that there were no inhibitors in the environmental samples. This confirms that the negative results obtained from environmental DNA were not due to the presence of inhibitors in the water sample but to the absence of *Potamopyrgus* DNA in the samples. Therefore false negatives were discarded.

### Phylogenetic inferences

From the individuals analyzed in this study a total of 26 haplotypes were found: 11 (two from Asturias *P*. *antipodarum* individuals), 8 (two from Asturias individuals) and 7 (also two from Asturias) for COI gene, long, and short 16S rDNA fragments respectively. The haplotypes obtained in this study are available in NCBI GenBank database with the accession numbers KU932989-KU932999 (COI gene), KU933003-KU933010 (16S rDNA large fragment). The shorter 16S rDNA amplicon obtained from taxon-specific primers corresponds to the sequence comprised between site 01 and site 325 on KU933003- KU933010.

**Fig 4:**
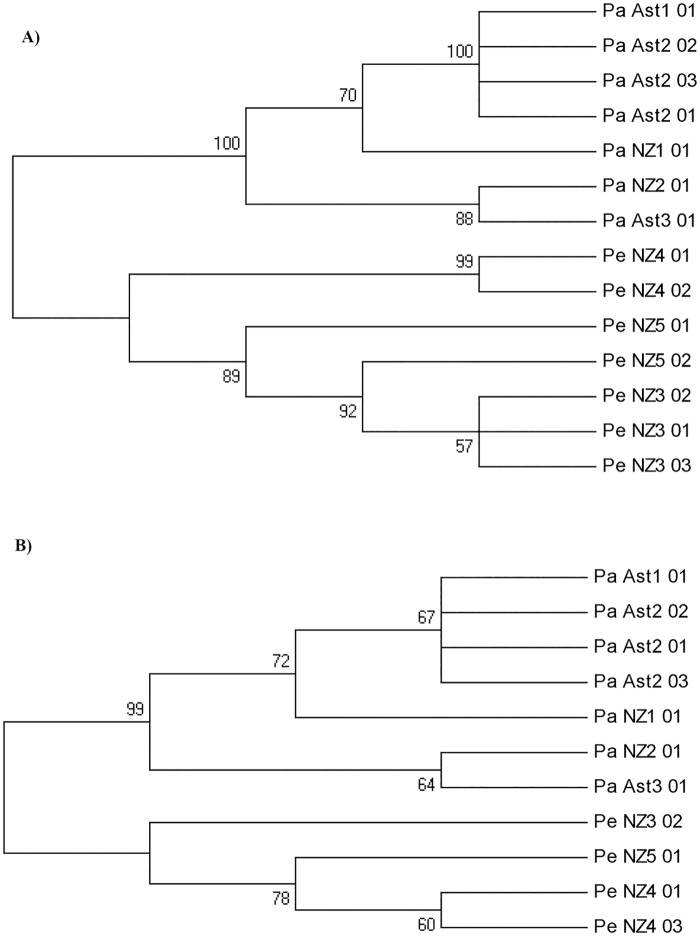
Phylogenetic tree reconstructed from: A) cytochrome oxidase I gene (621 nucleotides), and B) 16S rDNA haplotypes (496 nucleotides), obtained with universal primers [46, 47] from the individuals analyzed in this study. Pa and Pe are *Potamopyrgus antipodarum* and *P*. *estuarinus* respectively.

The tree reconstructed from the COI gene (Fig 4A) and 16S rDNA (Fig 4B) haplotypes obtained in our mud snail samples with universal primers separated consistently the samples from San Claudio (downstream) from those collected mid- and upstream. Downstream samples clustered with New Zealand samples (Onomalutu River) while the rest of River Nora samples, all with the same haplotype, clustered with River Collins samples, also from New Zealand. The *Potamopyrgus estuarinus* samples of Maitai River and the Matua Rangarawa Mangroves (New Zealand) clustered, as expected, in an independent branch for the two genes. They were separated by locations (Maitai River in one branch and Matua Rangarawa Mangroves in another) for 16S rRNA gene (Fig 4B), with apparent geographical differentiation.

**Fig 5:**
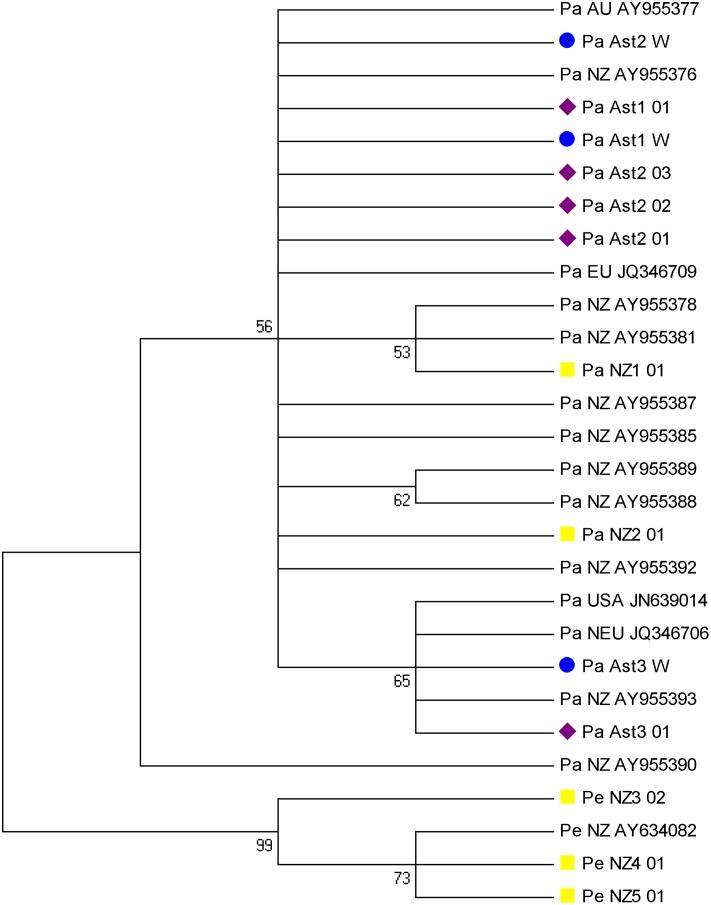
Phylogenetic tree of partial 16S rDNA sequence (325 nucleotides) with the taxon-specific primer reconstructed from the *Potamopyrgus* haplotypes (Pa, *P*. *antipodarum*; Pe, *P*. *estuarinus*) obtained in this work and references obtained from GenBank (the accession number is indicated). The geographic origin of the voucher *P*. *antipodarum* specimens are: Ast1; Ast2; Ast3; USA; NZ; NZ1; NZ2; NEU and EU are: Colloto, Lugones, S. Claudio (Asturias 1, 2, and 3), Wisconsin, New Zealand; Collins River, Onomalutu River (New Zealand, South island); Estonia, France (European samples). Sequences obtained from the water samples, Asturias individuals and New Zealand individuals (south island) sampled in this study are indicated with a blue circle, a purple diamond and a yellow square respectively.

For the shorter 16S rDNA fragment amplified with the primers designed herein, the two haplotypes of *P*. *antipodarum* found in Asturias (from both water samples and mud snail individuals) were also separated in different clusters (Fig 5). Samples from Lugones and Colloto (purple diamond in Fig 5) formed a monophyletic group with one haplotype (JQ346709) found in Germany, France, Hungary, Poland, Lithuania and United Kingdom; with the haplotype AY955377 found in Australia (Tasmania), and the New Zealand haplotype AY955376. The haplotype found downstream River Nora (PaAst3-03, San Claudio location) was in a separate clade supported by a bootstrap value of 65, containing the haplotype JN639014 found in Estonia and Wales; the haplotype JN639014 found in Hammond Harbor in Oregon and Devils Lake in Wisconsin; and the New Zealand haplotype AY955393 (North Island). The haplotypes of New Zealand South Island were also separated in this tree, the haplotype from River Collins being monophyletic with the upstream and midstream Asturian samples and other European references, and the Onomalutu River haplotype exhibiting an intermediate and less clear position in the middle of the two branches (Fig 5). Indeed the haplotypes obtained from Asturias water samples (blue circles in Fig 5, GenBank accession numbers KU933000-KU933002) matched perfectly with the haplotypes of the individuals found from the same place.

The *Potamopyrgus estuarinus* samples from New Zealand analyzed in this work formed a clearly differentiated clade with a *P*. *estuarinus* reference sequence from GenBank (AY634082), supported by a bootstrap value of 99. Since the two species are closely related, this confirms the phylogenetic value of this relatively short marker.

## Discussion

This is the first record of *Potamopyrgus antipodarum* from the central basin of the Bay of Biscay (Asturias, North of Spain). In the Iberian Peninsula the species has been detected in Atlantic and Mediterranean basins [16, 20], but not in the Bay of Biscay façade.

The results obtained in this study are surprising in several ways. First, in a small river (River Nora) and at short distance among sampling locations we have found two different haplotypes. These haplotypes correspond to the haplotypes *t* and *z* described for European *Potamopyrgus antipodarum* by Städler *et al*. [36]. These authors found the two haplotypes together only in two locations: Loch of Stennes (Orkney, Scotland) and Slack estuary (Nord-Pas de Calais, France). In the rest of sites studied across Europe only one haplotype was present in each location. The two haplotypes do not seem however be admixed in the same place. The sequences obtained from water samples did not exhibit any sign of overlapped chromatogram peaks in the polymorphic sites described by Städler *et al*. [36]. Low densities and scarce juveniles found downstream suggest that a second and recent introduction occurred in San Claudio site.

Another interesting result of this study was high sensitivity of the taxon-specific primers developed for detecting *Potamopyrgus* DNA in water samples. Goldberg *et al*. [35] designed a marker in the cytochrome b gene region that was able to detect *Potamopyrgus* individuals at densities as low as 11 individual/m^2^, filtering 4-L water samples. In our study successful amplification of the 16S rDNA based marker, with amplicons visible on agarose gel, was obtained from 1-L water samples and almost half density (6 individual/m^2^). This viviparous mud snail does not have a planktonic stage, so the DNA detected from water samples is most likely free-floating DNA.

These results are really encouraging because, since imply usefulness of this for early detection of the species when the population density is still low at the initial stage of invasion or on the edge of the range expansion area. This PCR method is economical (the estimated average cost was 10 euros per water sample) and faster in comparison to Metabarcoding [51], and also to qPCR [52] and could be easily added into routine surveillance programs.

The method has a shortcoming, however. Simple positive amplification and visualization in agarose gel (or by capillary electrophoresis), that can serve for detecting the species in Europe and North America because it is unique in its genus there, are not enough for population monitoring in its native settings. *In silico*, and proved *in vitro* for *P*. *estuarinus*, the primers can anneal with other species of the genus *Potamopyrgus* that are present in Australia and New Zealand. The DNA region employed here as a marker has the phylogenetic power to discriminate between closely related species of this genus (Fig 4). The same primers could be used in native settings using high throughput methodologies [53], or simply cloning-sequencing to separate the different amplicons. Since the region amplifies well from water samples, after further development it could be employed as an additional method for surveys of native *Potamopyrgus* species assemblages.

The origin of the *Potamopyrgus antipodarum* found in Asturias seems to be the same as for the rest of Europe, since the two haplotypes described by Städler *et al*. [36] were found. The particular introduction pathway to the region, however, is still unclear. Ballast water, one of the inferred vectors of this invader [13], can be reasonably discarded in our case because the invaded habitats are not accessible from the sea (isolated by an impassable dam). Upstream River Nalón we found no *P*. *antipodarum* individuals neither traces of its DNA in the water (negative controls). Aquaculture can also be disregarded because there are no aquaculture facilities in River Nora valley. Short-distance transport by fishermen as suggested by Alonso and Castro-Díez [16] is plausible. Casual hikers may contribute to short-distance transport as well. The bird-mediated transport suggested by Lassen [54] is also plausible, since the region is in the middle of the 600-km corridor of northern Spain that is an important and rich wintering ground for many birds [55, 56]. Another possibility, still unexplored, is that they could come from aquarium releases as accompanying fauna of fish pets, as already described for other species [57].

Loo *et al*. [58] predicted extremely fast spread of this species, forecasting a total invasion of North America freshwater ecosystem in a relatively short time if actions are not taken to prevent its expansion. It seems that in the Cantabrian range region, or at least in the river where it was detected for the first time, the population density is still not too high, especially downstream. Rapid application of containment measures and eradication efforts, as well as a close surveillance of the present populations could be strongly recommended.

## Conclusion

We developed a specific set of primers to detect *Potamopyrgus* species directly from the water samples (environmental DNA). With this molecular tool it is possible to establish the species identity and the phylogenetic characteristics of the invasion, sequencing PCR amplicons obtained from environmental samples. This powerful (and economical if limited to visualization on gel) tool can be useful for early detection of New Zealand mud snail in its expanded range of invasion.
